# Transcriptional Profiling of Testis Development in Pre-Sexually-Mature Hezuo Pig

**DOI:** 10.3390/cimb47010010

**Published:** 2024-12-29

**Authors:** Zunqiang Yan, Qiaoli Yang, Pengfei Wang, Shuangbao Gun

**Affiliations:** College of Animal Science and Technology, Gansu Agricultural University, Lanzhou 730070, China; yanzq@gsau.edu.cn (Z.Y.); yangql@gsau.edu.cn (Q.Y.)

**Keywords:** scRNA-seq, Hezuo pig, spermatogenesis, testis

## Abstract

Spermatogenesis is an advanced biological process, relying on intricate interactions between somatic and germ cells in testes. Investigating various cell types is challenging because of cellular heterogeneity. Single-cell RNA sequencing (scRNA-seq) offers a method to analyze cellular heterogeneity. In this research, we performed 10× Genomics scRNA-seq to conduct an unbiased single-cell transcriptomic analysis in Hezuo pig (HZP) testis at one month of age during prepuberty. We collected 14,276 cells and identified 8 cell types (including 2 germ cells types and 6 somatic cell types). Pseudo-timing analysis demonstrated that Leydig cells (LCs) and myoid cells (MCs) originated from a shared progenitor cell lineage. Moreover, the functional enrichment analyses showed that the genes of differential expression were enriched in spermatogonia (SPG) and were enriched in the cell cycle, reproduction, and spermatogenesis. Expressed genes in spermatocytes (SPCs) were enriched in the cAMP, cell cycle, male gamete generation, reproductive system development, and sexual reproduction, while growth hormone synthesis, gamete generation, reproductive process, and spermine synthase activity were enriched in Sertoli cells (SCs). Additionally, chemokine, B cell receptor, activation of immune response, and enzyme binding were enriched in macrophages. Our study investigated transcriptional alterations across different cell types during spermatogenesis, yielding new understandings of spermatogenic processes and cell development. This research delivers an exploration of spermatogenesis and testicular cell biology in HZP, establishing the groundwork for upcoming breeding initiatives.

## 1. Introduction

The Hezuo pig (HZP) is a local breed in Gansu Province that inhabits the Tibetan Plateau at elevations above 2500 m [1,2,3]. It is characterized by its dense fleece, which enables it to endure harsh conditions such as alpine hypoxia [3]. It serves as a crucial source of meat for the region and provides significant economic benefits to local herders [4,5]. Moreover, it plays essential roles in promoting socioeconomic development and maintaining pasture ecosystems [2,3]. Despite its many advantages, it has lower reproductive performance compared to other pig breeds [3,6]. Enhancing its reproductive capabilities, particularly focusing on male reproductive performance, is crucial for increasing production. Further studies on HZP testis could improve reproductive efficiency. Our previous studies found that it reaches puberty earlier than Landrace pig [3,6]. We analyzed the expression and characterization of lncRNAs and miRNAs in HZP testes, identifying many differentially expressed lncRNAs (such as *LOC110259451* and *LOC102166108*) and miRNAs (such as ssc-miR-199b, ssc-miR-383 and ssc-miR-149) associated with spermatogenesis and precocious puberty through ECM–receptor interaction, PI3K-Akt, TGF-beta, and MAPK pathways [6,7]. However, due to the vast number of cells within the testis, research on the regulatory mechanisms underlying testicular development in HZP, as well as the identification of associated regulatory molecules, remains highly limited. Unveiling the key regulatory molecules involved in testicular development holds significant potential for enhancing fertility, expanding the HZP population, and advancing the industrial development of this breed.

The testis is principally comprised of spermatogenic tubules and testicular mesenchyme [8]. The spermatogenic tubules consist of boundary membrane and seminiferous epithelium, which have various germ cells and Sertoli cells (SCs) [9,10]. The testicular mesenchyme is predominantly comprised of Leydig cells (LCs), myoid cells (MCs), and macrophages [11]. Spermatogenesis itself is a complex, meticulously regulated biological process within the seminiferous epithelium, where spermatogonia undergo differentiation into mature spermatozoa [9,12,13]. SCs stand out as among the most intricate in the body, endowed with a remarkable capacity to continually reshape themselves and adjust their functions to effectively coordinate spermatogenesis, providing physical support and nourishment to developing male germ cells [14,15]. In the basolateral extracellular matrix, between adjacent SCs, the blood-testis barrier is generated, effectively compartmentalizing the seminiferous epithelium into separate basal and adluminal regions [16,17]. The basal compartment is the reservoir for spermatogonia, while the adluminal compartment is dedicated to more advanced germ cells, creating a finely tuned microenvironment that facilitates meiosis and promotes the development of spermatids into spermatozoa [18,19]. LCs, dispersed within the testicular interstitium, are tasked with synthesizing testosterone, a hormone crucial for spermatogonial growth, meiotic development, and sperm release, as well as for exerting direct effects on other somatic cells within the testis [20,21,22,23]. MCs provide a foundation and produce peristaltic waves through their contractile components, aiding the motion of fluid within the tubule lumen to help discharge spermatozoa [23,24]. Therefore, SCs, LCs, and MCs constitute an intricate communication network, establishing a highly regulated microenvironment for germ cells, thereby orchestrating the process of sperm production, because preserving the functional integrity of the testis is important to sustaining spermatogenesis [12,25,26,27]. Nevertheless, the testis, characterized by its complexity and the presence of diverse germ and somatic cell types, presents considerable challenges in analyzing cell types across developmental stages and in profiling gene expression within specific cell populations. Traditional methods for studying testicular biology often face significant challenges, due to the cellular heterogeneity of the testis, particularly in identifying and analyzing cell-specific gene expression at different developmental stages. Recently, single-cell RNA sequencing (scRNA-seq) has become a valuable technique, allowing for the analysis of thousands of unique characteristics in individual cells and offering highly detailed transcriptomic maps of tissues and organs [28,29,30]. This approach has been widely applied to investigate spermatogenesis across various species, including buffalo [31], yak [32,33], goat [34,35], pig [36,37], sheep [38,39], and giant panda [40]. For example, Wu et al. conducted scRNA-seq to explore spermatogenesis of 3-month-old sheep, obtaining 12,843 testicular cells and identifying 9 testicular somatic cell types (such as MCs, LCs) and 5 germ cell types (such as spermatogonia (SPG) and spermatocytes (SPCs)), as well as identifying several specific marker genes in the germ cells at different states of differentiation [39]. Additionally, Zheng et al. characterized testicular cells in the giant panda using scRNA-seq, providing a detailed transcriptomic profile and identifying various cell types and significant pathways essential for giant panda reproduction, which enhances our knowledge of reproductive biology in endangered species [40].

Research on gametogenesis in HZP remains significantly underexplored; thus, we employed the scRNA-seq to examine gene expression profiles in testicular cells. Our analysis yielded expression profiles for 14,276 testicular cells, revealing 8 distinct cell types. Genes with differential expression were identified, and their functions were investigated. This paper advances our comprehension of the molecular mechanisms governing testicular development and spermatogenesis, establishing the groundwork for subsequent research on the breeding and conservation of HZP.

## 2. Materials and Methods

### 2.1. Ethics Statement

Experimental animals conformed to the guidelines founded by the Ministry of Chinese Agriculture. The Committee on Animal Ethics at GAU permitted procedures related to the animals (2006-398). Efforts were made to ensure minimal suffering.

### 2.2. Sample Collection and HE Staining

HZP primarily graze on hillsides, river valleys, and harvested wheat fields, consuming wild plants like stems, leaves, seeds, and rhizomes. They receive minimal supplementary feed, and drink water regularly. Testis was achieved from a one-month-old male HZP, which was euthanized via the injection of pentobarbital sodium solution [41]. The testis was rinsed three times with DPBS (Gibco, Waltham, MA, USA), chopped into small sections and transferred into cryotubes filled with a cryopreservation medium containing 70% DMEM (Invitrogen, Carlsbad, CA, USA) + 20% FBS (Gibco) + 8% DMSO (Sigma-Aldrich, St. Louis, MO, USA) + 2% penicillin-streptomycin (Gibco). The sample was subsequently slow-frozen, stored in liquid nitrogen, and used to prepare single-cell suspensions.

Following previously established methodologies [7], the testicular tissue underwent sectioning and staining. Small fragments of testicular tissue were prepared and fixed in a diluted Bouin’s solution for 72h before being embedded in paraffin. Samples were then sliced into 5 µm thick paraffin sections, which underwent deparaffinization and rehydration. Finally, hematoxylin and eosin were used to treat sections for evaluating histological structure and morphology.

### 2.3. Preparation of Single-Cell Suspensions

Frozen samples were defrosted quickly at 37 °C. After thawing, fragments were treated with type IV collagenase at 37 °C for eight minutes, followed by two washes with cold DPBS. Next, 0.25% trypsin-EDTA and 0.25 mg/mL DNase I (Sigma-Aldrich) were added, and the mixture was incubated at 37 °C for ten minutes. Digestion was terminated with the addition of DMEM medium supplemented with 10% FBS. Single testicular cells were then counted and resuspended in PBS with 0.4% bovine serum albumin for scRNA-seq.

### 2.4. 10× Genomics Library Preparation and Sequencing 

Following cell suspension preparation, 0.4% trypan blue solution was used to evaluate cell viability. The concentration of viable cells was subsequently adjusted to 1500–2000 cells/mL. The cell suspension was then processed using 10× Genomics technology for cell capture and library preparation by the Gene Denovo (Guangzhou, China). The final library was sequenced using the PE150 mode of the Illumina sequencing platform (NovaSeq 6000). The detailed library preparation and sequencing referred to the published academic paper [31].

### 2.5. Genome Alignment and Gene Expression Quantification

Barcode processing and single-cell 3’ gene counting was performed using Cell Ranger (v3.1.0) software. This process converted raw BCL files into FASTQ format, aligned the reads, and quantified the counts. Reads with low-quality barcodes or unique molecular identifiers (UMIs) were excluded. Then, the FASTQ files were mapped to the pig reference genome (*Sus scrofa* 11.1) by STAR RNA-Seq aligner [42]. Once aligned, barcodes associated with these read UMIs were subjected to filtering and correction. And, only reads uniquely aligned to the transcriptome with at least 50% exon overlap were considered valid UMIs. UMI sequences were corrected for sequencing errors, and the EmptyDrops method [43] was applied to identify valid barcodes. The resulting UMI counts and cell barcodes were used to construct cell-by-gene matrices. The methods for detailed genome alignment and quantification of gene expression were based on the protocols described in the referenced publication [31].

### 2.6. Cell Clustering and DGE Annotation

The Seurat (v3.1.1) software [44] was utilized to handle the matrices. Initially, filtering was performed according to three criteria: (I) the mitochondrial gene percentage was limited to under 10%; (II) a minimum of 200 genes had to be detectable per cell; (III) each gene was required to be expressed in at least three cells. Canonical correlation analysis (CCA) was employed to standardize and refine the gene-barcode matrix, thereby reducing the dimensionality of features. The resulting components were utilized for t-distributed stochastic neighbor embedding (t-SNE), enabling visualization of clusters in a two-dimensional space. Cells exhibiting similar expression profiles were grouped using a graph-based clustering algorithm.

Differential expression analysis was conducted via the Wilcoxon rank sum test [45] using the gene expression matrices filtered through Seurat [44] to identify differentially expressed genes (DEGs) for each cell cluster. Significantly unregulated genes were screened according to three standards [31]: (1) log2(fold change [FC]) ≥ 1.28; (2) the percentage of cells where the gene is detected in a specific cluster > 25%; and (3) *p*-value ≤ 0.05. Functional enrichment of DEGs within each cluster was then examined.

### 2.7. Cell Trajectory Analysis

Pseudo-time analysis was utilized to explore the differentiation trajectories of cells and the progression of cell subtypes. This technique arranges cells along a pseudo-time continuum on the basis of the expression profiles of critical genes, allowing for progressive shift modeling that occurs throughout development. The pseudo-time analysis was conducted via the Monocle2 software [46].

## 3. Results

### 3.1. Overview of scRNA-seq Data and Histomorphological Analysis of Testis

**Figure 1:**
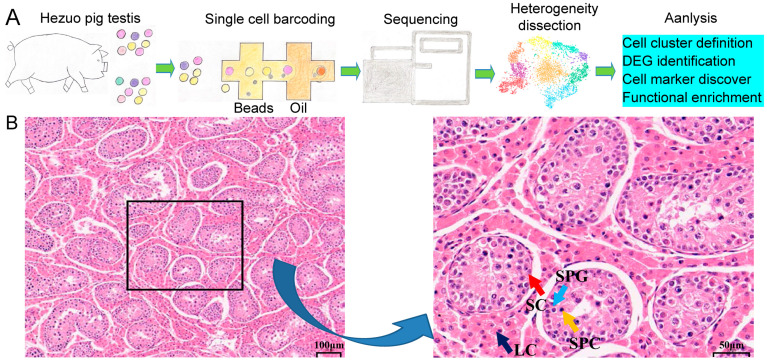
(**A**) A schematic representation of the experimental workflow. (**B**) Histological examination. Left panel: magnified 100×; right panel: magnified 400×.

To investigate the cellular composition, a sample was collected from HZP through veterinary surgery. The testis was fragmented, cryopreserved in liquid nitrogen, and subsequently thawed. Following enzymatic dissociation, scRNA-seq was conducted. The data analysis encompassed cell cluster identification, DEG screening, cell marker discovery and functional enrichment analysis (Figure 1A). Furthermore, as indicated in Figure 1B, the testicular tissue was intact, the seminiferous tubules were loosely arranged, and the lumen was small. A small number of SPG, SPCs, and SCs were found in the seminiferous epithelium. However, no spermatid and sperm were found. The testicular interstitium was a loose connective tissue, rich in blood and lymphatic vessels. We observed a large number of testicular LCs with a large cell volume and rich cytoplasm.

**Figure 2:**
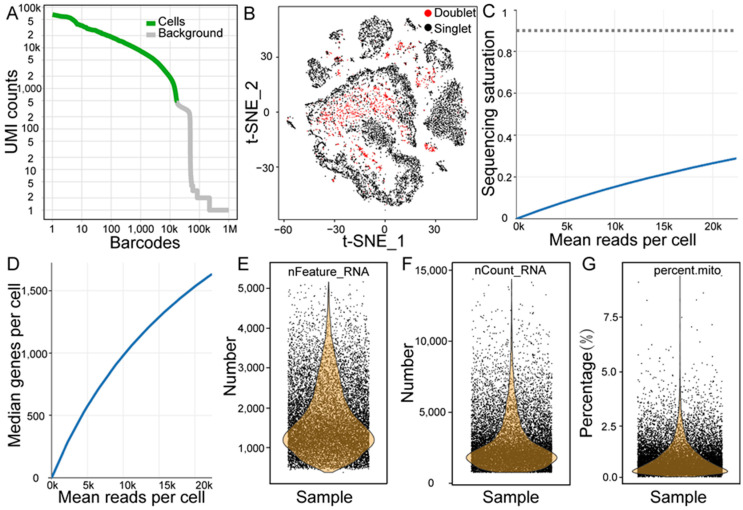
Data quality overview. (**A**) Visualization of effective cell detection. (**B**) Multicellular clustering displayed using the t-distributed stochastic neighbor embedding (t-SNE) diagram. (**C**) Sequencing saturation depicted on a map. (**D**) Median gene count per cell. (**E**) Distribution of detected gene numbers. (**F**) Distribution of total unique molecular identifier (UMI) counts. (**G**) Percentage of mitochondrial gene expression across individual cells.

A total of 16,355 testicular cells were acquired, with 14,276 analyzed (Figure 2A) after applying filtering criteria, including the eradicating double cells (Figure 2B; Table S1). The library generated 366.62 million reads with a sequencing saturation rate of 28.9% (Figure 2C). A total of 94.7% of reads were successfully mapped to the pig reference genome, with 90.9% showing high confidence. Additionally, Q30 was obtained in 95.9%, 90.5%, and 95.1% of the base reads in the barcodes, RNA, and unique molecular identifiers (UMIs). In total, 24,935 genes were detected, with a median number of genes of 1634 (Figure 2D). The quality metrics of testicular cells were assessed (Figure 2E–G and Table S2), revealing a median of 2274 UMI counts per cell and a mitochondrial UMI ratio of about 0.85%.

### 3.2. Determination of Testicular Cell Types Using Cluster Analysis

**Figure 3:**
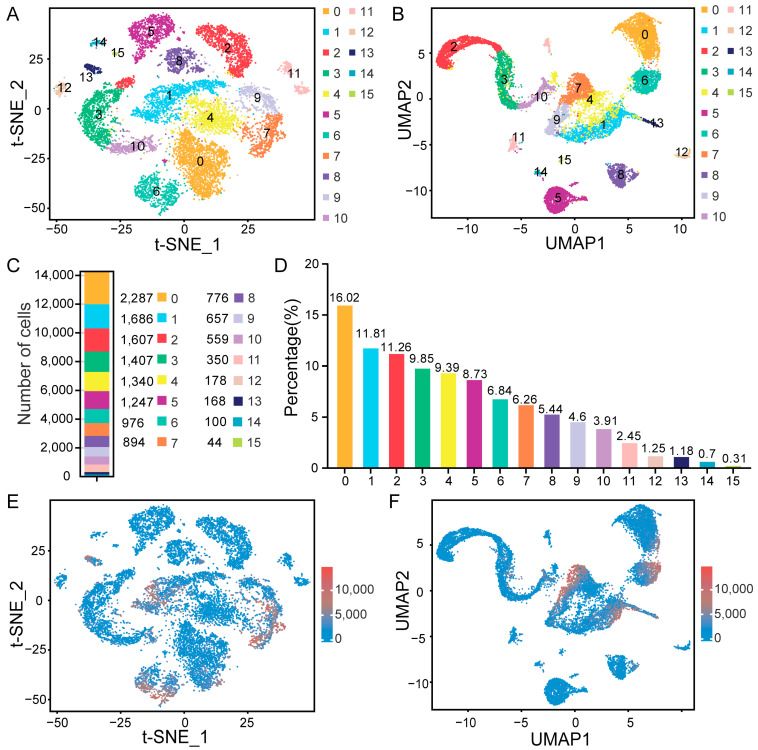
Transcriptome profile and cluster analysis of testicular cells. (**A**) t-SNE plot showcasing the clustering of unselected spermatogenic cells. (**B**) Uniform manifold approximation and projection (UMAP) plot displaying the profiling of spermatogenic cells. (**C**) Stacked bar chart indicating cell counts in each cluster. (**D**) Bar chart representing the proportion of cells across 16 clusters. (**E**) t-SNE and (**F**) UMAP plots displaying transcript expression level via UMIs.

**Figure 4:**
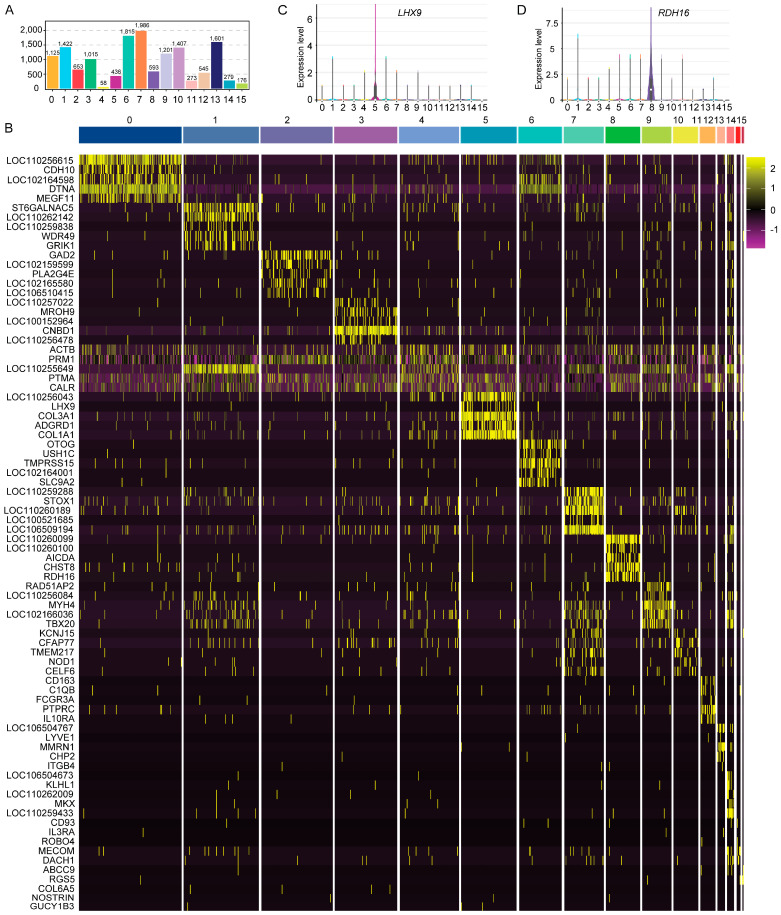
Analysis of differentially expressed genes (DEGs). (**A**) The count of DEGs identified within every cluster. (**B**) Heatmap illustrating a total of 80 DEGs across the various clusters. (**C**,**D**) Violin plots depicting the expression trend of *LHX9* and *RDH16* genes.

To mitigate the substantial technical noise, we applied t-SNE and uniform manifold approximation and projection (UMAP) analyses to the scRNA-seq expression matrix. Through these analyses, clustering according to gene expression trend revealed 16 distinct cell clusters (Figure 3A,B). The number of cells and their corresponding percentages in each cluster are presented (Figure 3C,D). Cluster 0 had the highest number of cells, at 2287, representing 16.02%, while cluster 13 had the lowest count of cells value, at 44, accounting for just 0.31%. Additionally, the majority of cells exhibited low levels of transcript expression (Figure 3E,F). In this study, cluster 7 exhibited the highest number of DEGs, at 1986, while cluster 4 had the fewest number of DEGs, at 58, respectively (Figure 4A). The top five DEGs for each cluster were identified and visualized using a heat map (Figure 4B). To validate these findings, two DEGs (including *LHX9* and *RDH16*) were randomly chosen to assess expression level (Figure 4C,D). As anticipated, these DEGs demonstrated the peak expression level in their corresponding clusters.

**Figure 5:**
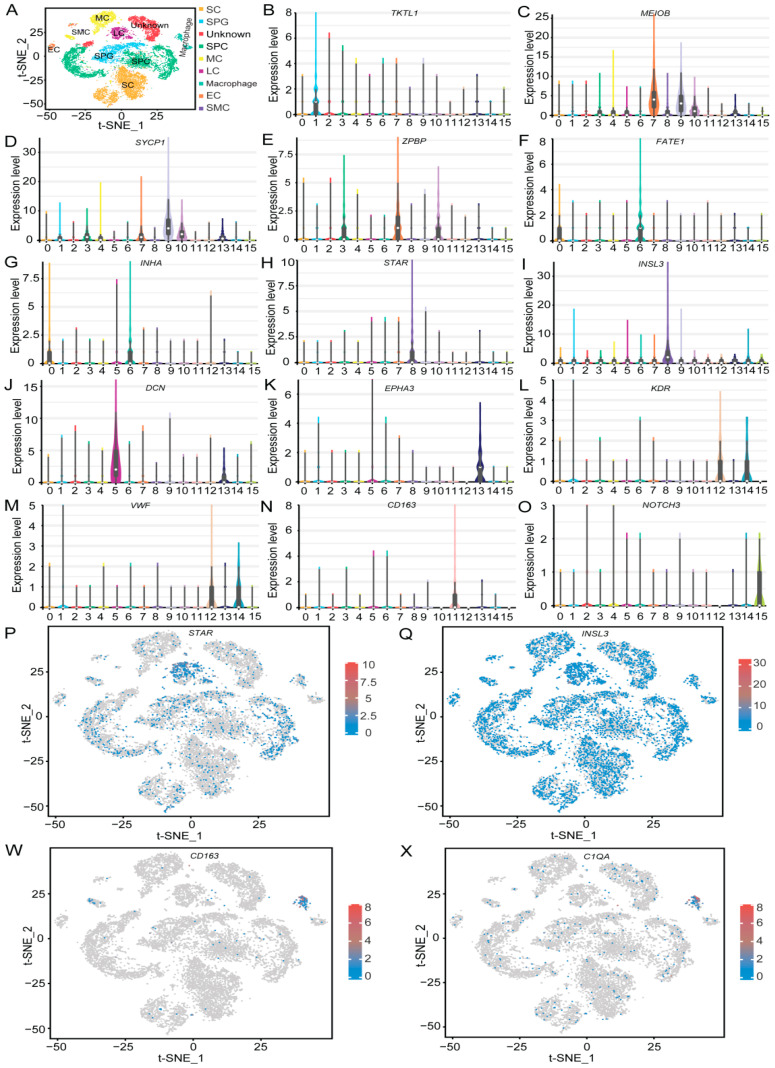
Detection of cell types. (**A**) t-SNE plot showing cell-kind identification. (**B**–**O**) Violin plots presenting the expression of cell type-specific genes in various clusters. (**P**,**Q**,**W**,**X**) t-SNE plots showing the expression pattern of *STAR*, *INSL3*, *CD163* and *C1QA* gene across different clusters.

Without specific germ cell marker genes for HZP, we used general marker genes from other species to classify cell categories in each cluster, based on their gene expression profiles. By leveraging these established cell type-specific markers, we identified eight distinct testicular cell subsets involved in spermatogenesis (Figure 5A). Cluster 1 cells were characterized by the expression of SPG marker genes *TKTL1*, *KIT*, and *UCHL1* (Figure 5B, Figure S1A,B). Clusters 3, 4, 7, 9, and 10 were associated with SPCs marker genes *MEIOB*, *SYCP1*, *ZPBP*, *DMRTC2*, *SYCE1*, *RAD51AP2*, and *NME8* (Figure 5C–E, Figure S1C–F). Clusters 0 and 6 were predominantly expressed SCs marker genes *FATE1*, *INHA*, *CLDN11,* and *CLU* (Figure 5F,G, Figure S1G–H). Cluster 8 was obviously expressed LCs marker genes *STAR*, *INSL3*, *APOE*, *CYP11A1*, and *CYP17A1* (Figure 5H,I, Figure S1I–K). Clusters 5 and 13 were highly expressed MCs marker genes *DCN*, *EPHA3*, *PDGFRA*, and *PTCH1* (Figure 5J,K, Figure S1L,M). Clusters 12 and 14 were characterized expressed ECs marker genes *KDR*, *VWF*, and *PECAM1* (Figure 2L,M, Figure S1N). Macrophages marker genes *CD163* and *C1QA* were found in cluster 11 (Figure 5N, Figure S1O). Lastly, cluster 15 was the extremely expressed SMCs marker gene *NOTCH3* (Figure 5O). The cell types in cluster 2 remain undefined.

**Figure 6:**
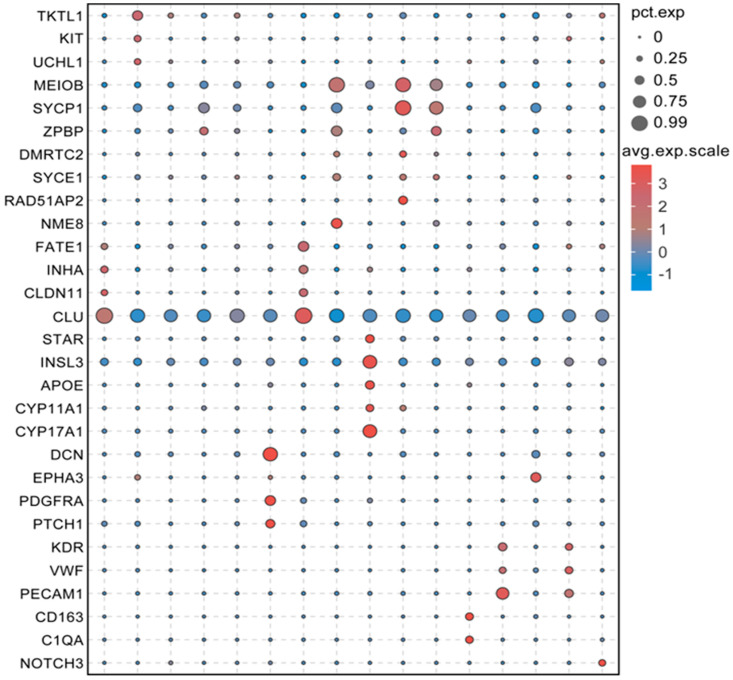
Dot plots displaying the expression pattern of cell-specific genes in testicular cells.

The expression pattern of these marker genes was visualized using a bubble chart, clearly demonstrating the distinct separation of all clusters (Figure 6). The heatmap further revealed that certain marker genes tended to cluster together (Figure S2). Specifically, the LCs marker genes include *STAR*, *INSL3*, *APOE*, *CYP11A1*, and *CYP17A1*. Additionally, the t-SNE plots demonstrated that the selected marker genes were expressed in every cluster (Figure 5P–X, Figure S3). For example, *STAR* (Figure 5P) and *INSL3* (Figure 5Q) displayed elevated expression levels in LCs (Cluster 8), where they are associated with testosterone production, while their expression was notably lower in other clusters. Similarly, *CD163* (Figure 5W) and *C1QA* (Figure 5X) showed higher expression levels in macrophages (cluster 11), correlating with immune response functions, but were expressed at lower levels in other clusters.

### 3.3. MCs and LCs Derived and Differentiated from a Shared Progenitor Lineage

**Figure 7:**
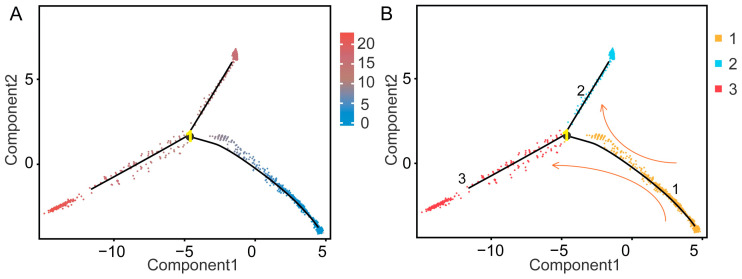
Pseudo-time analysis of Leydig cells (LCs) and myoid cells (MCs). Pseudo-time data (**A**) and differentiation status (**B**) of clusters 5, 8, and 13 suggested a shared progenitor for the LC and MC lineages. The pseudo-time scale represents the developmental progression, where lower values correspond to earlier stages. Different colors highlight distinct stages of differentiation.

Previous studies researching human, goat and yak testes, have noted that LCs and MCs originate from the same progenitor cell group [32,35,47]. To explore whether LCs and MCs in HZP share a common progenitor, clusters 5, 8, and 13 were subjected to pseudo-time analysis. Our analysis confirmed that LCs and MCs differentiate from a shared progenitor lineage (Figure 7).

### 3.4. Analysis of Functional Enrichment in Germ Cells

**Figure 8:**
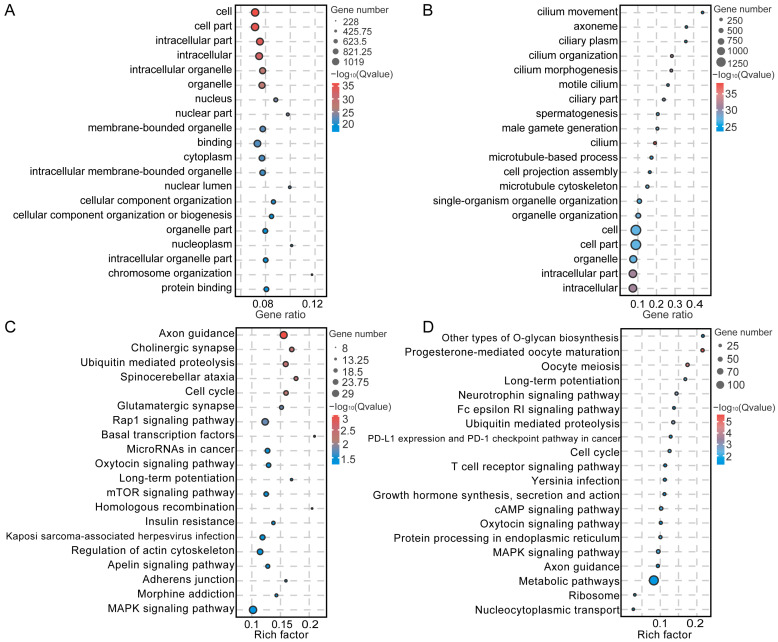
Functional enrichment analysis of spermatogonia (SPG) and spermatocytes (SPCs). (**A**,**B**) represent the top 20 GO terms for SPG and SPCs DEGs, while (**C**,**D**) illustrate the top 20 KEGG pathways for SPG and SPCs DEGs.

Spermatogenesis is characterized by complex and organized transformations in spermatogenic cells. To investigate the distinctions between SPG and SPCs, the DEGs were analyzed using the GO and KEGG annotation databases. In the biological process (BP) classification, the DEGs in SPG were primarily associated with spermatogenesis, regulation of gene expression, DNA conformation change, spermatogonial cell division, reproduction, chromatin remodeling, and spermatid nucleus elongation; within the cellular component (CC) classification, they were mainly related to the nucleus, sperm principal piece, and sperm part; for the molecular function (MF) classification, they were associated with chromatin binding, and ATP binding (Figure 8A, Table S3). In SPCs, the DEGs demonstrated enrichment in male gamete generation, spermatogenesis, cilium movement, reproduction, reproductive system development, meiotic cell cycle, and sexual reproduction in the BP classification; in the CC classification, they showed enrichment in cilium, sex chromosome, and sex chromatin, while in the MF classification, they were related to nucleic acid binding, and anion binding (Figure 8B, Table S4). The DEGs in SPG were primarily associated with cell cycle, the axon guidance, cholinergic synapse, and ubiquitin-mediated proteolysis pathways (Figure 8C, Table S3). Meanwhile, the DEGs in SPCs were notably related to cAMP, cell cycle and spliceosome pathways (Figure 8D, Table S4).

### 3.5. Analysis of Functional Enrichment in Somatic Cells

**Figure 9:**
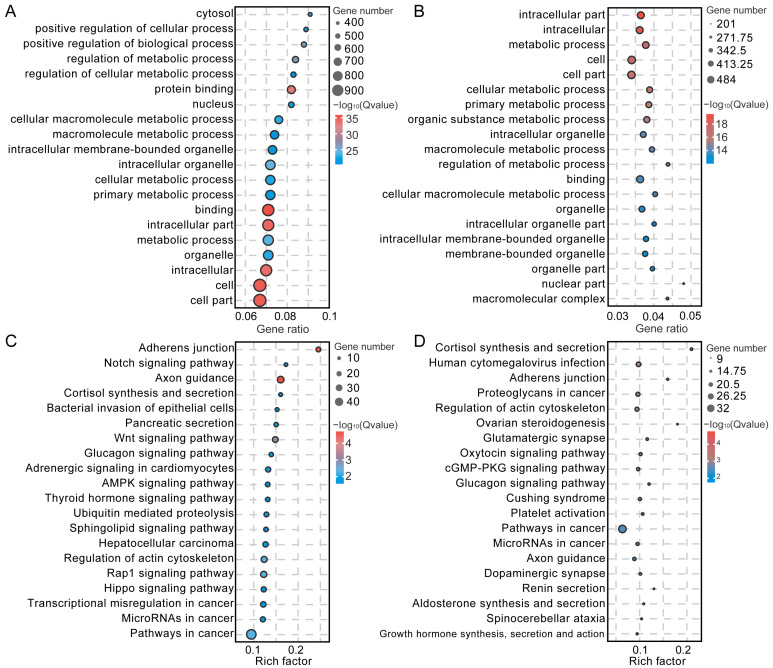
Functional enrichment analysis of Sertoli cells (SCs) and LCs was performed. The top 20 GO terms associated with DEGs in SCs (**A**) and LCs (**B**) are presented. Additionally, the top 20 KEGG pathways linked to DEGs in SCs (**C**) and LCs (**D**) are identified.

Somatic cells within the testes are essential for the processes of testicular development and sperm production. As a result, GO-term functional enrichment analysis was conducted on six identified testicular cell types to examine their functions. In SCs, the DEGs were associated with regulation of metabolic process, gamete generation, sexual reproduction, and reproductive process in the BP classification; for the CC classification, they were enriched in nucleus, cytoplasm, spermatoproteasome complex, sperm part, and cytoplasm; while in the MF classification, they showed enrichment in protein binding, and spermine synthase activity (Figure 9A, Table S5). In LCs, the DEGs were linked to cellular metabolic process, spermatid nucleus differentiation, and sperm chromatin condensation within the BP classification; for the CC classification, they were enriched in nuclear part, spermatoproteasome complex, and sperm part; under the MF classification, enrichment was observed in binding, and enzyme binding (Figure 9B, Table S6). The DEGs in MCs showed enrichment in the BP classification, particularly in reproductive system development and the reproductive process; within the CC classification, they were associated with sperm-head plasma membrane, sperm midpiece, and synaptic membrane; in the MF classification, the DEGs were enriched in ion gated-channel activity and structural molecule activity (Figure S4A, Table S7). In ECs, the DEGs were enriched in the BP classification, particularly in cell adhesion, spermatogenesis, gamete generation, and sperm motility; for the CC classification, enrichment was observed in cell junction, sperm principal piece, cell periphery, and sperm part; within the MF classification, the DEGs were associated with protein binding, and protein kinase binding (Figure S4B, Table S8). In macrophages, the DEGs were associated with activation of immune response and cell activation under the BP classification; in the CC classification, they were enriched in sperm midpiece, sex chromosome, and intracellular part; for the MF classification, the DEGs demonstrated enrichment in enzyme binding and receptor binding (Figure S5A, Table S9). In SMCs, the DEGs were linked to cell migration, cell development, spermathecum morphogenesis, and spermatogenesis under the BP classification; within the CC classification, they were enriched in sperm part and sperm annulus; in the MF classification, they were associated with protein binding, cAMP binding, and actin binding (Figure S5B, Table S10).

We further conducted KEGG functional enrichment analysis for six somatic cell classifications. The DEGs of SCs were prominently enriched in thyroid hormone, AMPK, Wnt, regulation of actin cytoskeleton, growth hormone synthesis, secretion and action pathways (Figure 9C, Table S5). In LCs, the DEGs were primarily associated with adherens junction, steroid biosynthesis, GnRH, and MAPK pathways (Figure 9D, Table S6). For MCs, the DEGs were linked to ECM–receptor interaction, calcium, PI3K-Akt and cGMP-PKG pathways (Figure S4C, Table S7). The DEGs of ECs indicated significant enrichment in the focal adhesion, glutamatergic synapse, and tight-junction pathways (Figure S4D, Table S8). The DEGs in macrophages were enriched in Th17 cell differentiation, B cell receptor, viral myocarditis, and chemokine pathways (Figure S5C, Table S9). The DEGs of SMCs displayed enrichment in gap junction, vascular smooth-muscle contraction, and bacterial invasion of epithelial cell pathways (Figure S5D, Table S10).

## 4. Discussion

The testis serves as the primary reproductive organ, and its physiological integrity is essential for efficient sperm production [27,48,49]. The preservation of male fertility throughout life requires an uninterrupted process of spermatogenesis, governed by complex interactions between germ cells and surrounding somatic cells [15,50,51]. Conventional methods for analyzing the transcriptome of testicular cells have largely centered on the entire testis [52]. This approach is constrained by the cellular complexity of the testis, making it challenging to achieve high-resolution insights into gene expression specific to germ and somatic cells [52]. To address this challenge, methods such as FACS and MACS have been employed to isolate specific cell populations in large quantities [53]. Nevertheless, the advent of scRNA-seq has gained considerable attention for its ability to analyze cellular heterogeneity with a smaller cell requirement [54]. High-dimensional scRNA-seq has become recognized as a useful tool for studying the processes of development and differentiation across a wide range of sperm cell populations [28,54]. Utilizing scRNA-seq, numerous studies have explored both consistent and differential gene expression of testicular cells [32,36,37,39].

The distinct transcriptomes of HZP testicular cells are not yet well characterized. To address this knowledge gap, our study utilized scRNA-seq to investigate the cellular diversity within the testis. Utilizing the widely recognized 10× Genomics platform, we analyzed 14,276 cells from the testis of a one-month-old pig, successfully identifying critical germ and somatic cell types, based on established marker genes from mouse, human, and various mammals. To deepen our understanding, we identified DEGs for each cell type and performed functional enrichment analysis. LCs and MCs in the dairy goat testis, as demonstrated by Yu et al., revealed that both cell types share a common progenitor by Yu et al [35]. Similarly, Wang et al. demonstrated that LCs and MCs in yak testis cluster together and share a progenitor lineage [32]. To assess whether this phenomenon occurs in HZP testis, we conducted cluster analysis on testicular cells and observed that LCs and MCs formed a cohesive cluster, which is consistent with the previous observations [32,35].

In the mammalian testis, spermatogenic development and the continuous process of spermatogenesis are dependent on various testicular somatic cells [12,25,55]. SCs are crucial for providing structural and nutritional support, and maintaining testicular homeostasis in germ cell differentiation [15,17,19]. LCs are responsible for the synthesis of testosterone to regulate spermatogenesis, IGF1 and CSF1 secretion [21,22]. MCs secrete various growth factors to modulate the SCs function, to influence spermatogonia differentiation, and macrophages are essential in maintaining testicular homeostasis [11,24,50]. Consequently, numerous studies have employed scRNA-seq to explore the diverse somatic cell types in animal testes. For example, Zheng et al. characterized SCs, LCs, MCs, ECs, macrophages, and lymphocytes as the primary somatic cell types in the giant panda [40]. Zhang et al. reported the presence of SCs, LCs, MCs, ECs, macrophages, and lymphocytes in the Guanzhong black pig [37]. Tang et al. found SCs, LCs, MCs, ECs, macrophages, lymphocytes, and SMCs in the Shaziling pig [36]. In our study, we also identified these cell types (such as SCs, LCs, SMCs, and macrophage). Consistent with the aforementioned studies, most testicular somatic cells were identified; however, lymphocytes and natural killer cells were not detected.

The identification of somatic cell types in the HZP using established marker genes from species such as cattle and goats highlights the conserved gene expression trend in testes [30,56]. Supporting this, GO functional enrichment and KEGG pathway analyses demonstrated overlapping biological processes and functions across these cell types in different mammalian species [35,37,57,58]. The pivotal roles of somatic cells have promoted numerous reports focusing on functional enrichment analysis of DEGs in testicular somatic cells across various types of livestock. Wang et al. [32] revealed that in the yak testis, DEGs in SCs were predominantly related to cytosol, nucleus and extracellular-region GO terms, and were also implicated in cAMP and thyroid hormone synthesis pathways; LCs were linked to cytosol, nucleus, and cell adhesion GO terms, as well as PI3K-Akt Pathway in cancer, and ECM–receptor interaction pathways. Similarly, Tang et al. [36] demonstrated that in the Shaziling pig testis, DEGs in ECs were enriched in cell adhesion and endothelium-development GO terms, macrophages were associated with immune-response GO terms, and SMCs were linked to the cell-adhesion GO term. To gain a clearer understanding of the involvement of testicular somatic cells in HZP spermatogenesis, functional enrichment analyses were performed on DEGs from these cell types. In our study, SCs were mainly enriched in cytosol, and developmental-process and protein-binding GO terms, and were involved in cAMP, Wnt pathways; LCs were associated with developmental-process and protein-binding GO terms and thyroid hormone and Wnt pathways, highlighting their pivotal role in testosterone synthesis, which is essential for spermatogenesis in HZP; SMCs were involved in cell-adhesion and protein-binding GO terms; ECs were enriched in cell-adhesion and cell-periphery GO terms, while macrophages were associated with response to cytokine, immune-response GO terms, along with chemokine, bacterial invasion of epithelial cells pathways, underscoring their importance in combating microorganisms and preserving testicular homeostasis in the testis of HZP; moreover, MCs were enriched in tissue development and synaptic membrane GO terms, indicating their significant role in maintaining homeostasis. Indeed, numerous GO terms and pathways also identified in this paper aligned with previously reported findings [32,36,39]. While exploring the functional roles of testicular somatic cells exhibits slight variations across species, their primary function remains to support the process of sperm production.

Our study focused on identifying SPG and SPCs, utilizing established marker genes. In contrast to findings in the Guanzhong black pig [53], no additional SPG subtypes were detected, a result consistent with studies conducted on yaks [32]. Furthermore, spermatids and sperm were not observed, consistent with research on pre-sexual-maturity sheep [39], and this may be due to the use of testis from the pre-sexual maturity (one-month-old) HZP in our study. Notably, we identified several genes, including *LOC110256615*, *LOC102164598*, *LOC110262142*, and *LOC110259433*, which exhibit significant expression but have yet to be functionally characterized or reported across diverse cell types. Among these, *LOC110256615* and *LOC102164598* are predominantly expressed in SCs (cluster 0), *LOC110262142* is highly expressed in SPG (cluster 1), and *LOC110259433* demonstrates elevated expression in MCs (cluster 13). Indeed, these newly identified genes might contribute to spermatogenesis and act as a marker to identify testicular cell categories. Of course, further experiments are required to validate the reliability and scientific relevance of these newly identified genes. In this study, DEGs in SPG were intricately involved in regulation of cell development, DNA replication, chromatin remodeling, nucleus, nucleoplasm, protein binding, ATP binding, chromatin binding and organic cyclic compound binding. Indeed, these GO terms were consistent with findings reported in Shaziling pig testis [36], yak testis [32], goat testis [35] and sheep testis [39]. For example, DEGs in SPG of Shaziling pig were associated with DNA metabolic process, DNA repair and replication, and chromatin remodeling. DEGs in SPG of yak were related to DNA binding, nucleus, and ATP binding. Compared with these studies, our paper also found that some GO terms (such as reproduction, spermatid nucleus elongation, and sperm principal piece) were associated with testicular development and spermatogenesis. DEGs in SPCs were primarily enriched in GO terms, such as male gamete generation, spermatogenesis, reproductive system development, meiotic cell cycle, sexual reproduction, cilium and nucleus, which were consistent with findings in yak [32] and sheep [39] studies. For example, DEGs in SPCs of the Shaziling pig were associated with gamete generation and the meiotic cell cycle. DEGs in SPC of sheep were related to spermatogenesis and male gametogenesis. Additionally, significant enrichment of DEGs in SPG and SPCs was observed in cell cycle, cAMP, spliceosome, PI3K-Akt and ubiquitin-mediated proteolysis pathways, highlighting their crucial role in meiosis, which is consistent with research on yak and sheep testis [32,39]. Therefore, the germ cells were primarily associated with GO terms and KEGG pathways related to transcription and germ cell migration, providing further evidence of the proliferation and differentiation of germ cells during the pre-sexual period.

Our research provides a comprehensive classification of testicular cell groups in HZP. We successfully identified primary cell types and elucidated associated biological processes, paving the way for future research. This dataset and analysis are expected to offer valuable insights into male reproduction, ultimately contributing to the genetic enhancement and preservation of this breed. However, due to the high cost of single-cell samples, this study used only one sample, which was collected from pre-mature testicular tissue of a single pig breed. Consequently, the findings have certain limitations, as the mechanism of testicular development cannot be fully elucidated. To address these limitations, future research will include multiple pig breeds, additional developmental stages, and a larger number of samples, to obtain more comprehensive and reliable results.

## 5. Conclusions

Using scRNA-seq analysis and marker gene expression, we identified eight unique cell types within the testis. Among these, two were germ cell types, while the other six were somatic cell types. DEGs were identified for each cell type and further analyzed for functional enrichment. Our results indicated a shared progenitor cell origin for LCs and MCs. This dataset provides valuable insights into spermatogenesis in HZP, laying a foundation for pinpointing critical molecular markers essential to male germ cell development.

## Data Availability

The data are included in the article and its supplementary materials.

## Supplementary Materials

The following supporting information can be downloaded at: https://www.mdpi.com/article/10.3390/cimb47010010/s1. Figure S1: Violin plots showing the distribution of selected marker genes across different clusters. Figure S2: A heatmap of cell-specific genes in pig testicular cells. Figure S3: t-SNE plots of cell-specific marker genes in each cluster. Figure S4: GO and KEGG enrichment analysis for MCs and ECs. Figure S5: GO and KEGG enrichment analysis for macrophages and SMCs. Table S1: Details for each single cell. Table S2: Summary of data metrics. Table S3: Functional enrichment analysis of SPG. Table S4: Functional enrichment analysis of SPCs. Table S5: Functional enrichment analysis of SCs. Table S6: Functional enrichment analysis of LCs. Table S7: Functional enrichment analysis of MCs. Table S8: Functional enrichment analysis of ECs. Table S9: Functional enrichment analysis of macrophages. Table S10: Functional enrichment analysis of SMCs.

## References

[1] Tang Y., Zhang B., Shi H., Yan Z., Wang P., Yang Q., Huang X., Gun S. (2023). Molecular characterization, expression patterns and cellular localization of *BCAS2* gene in male Hezuo pig. Peerj.

[2] Wang L., Wang P., Yan Z., Zhang P., Yin X., Jia R., Li Y., Yang J., Gun S., Yang Q. (2024). Whole-plant silage maize to improve fiber digestive characteristics and intestinal microbiota of Hezuo pigs. Front. Microbiol..

[3] Yan Z., Song K., Wang P., Gun S., Long X. (2023). Evaluation of the genetic diversity and population structure of four native pig populations in Gansu Province. Int. J. Mol. Sci..

[4] Yan J., Wang P., Yan Z., Yang Q., Huang X., Gao X., Li J., Wang Z., Gao Y., Gun S. (2023). Cloning of *STC-1* and analysis of its differential expression in Hezuo pig. Anim. Biotechnol..

[5] Yin X., Wang P., Yan Z., Yang Q., Huang X., Gun S. (2024). Effects of whole-plant corn silage on growth performance, serum biochemical indices, and fecal microorganisms in Hezuo pigs. Animals.

[6] Zhang B., Yan Z., Wang P., Yang Q., Huang X., Shi H., Tang Y., Ji Y., Zhang J., Gun S. (2021). Identification and characterization of lncRNA and mRNA in testes of Landrace and Hezuo boars. Animals.

[7] Zhang B., Yan Z., Gao Y., Li J., Wang Z., Wang P., Yang Q., Huang X., Gun S. (2022). Integrated analysis of miRNA and mRNA expression profiles in testes of Landrace and Hezuo boars. Front. Veter Sci..

[8] Li L., Lin W., Wang Z., Huang R., Xia H., Li Z., Deng J., Ye T., Huang Y., Yang Y. (2024). Hormone regulation in testicular development and function. Int. J. Mol. Sci..

[9] Karimi H., Saraskanroud M.R., Koucheh F.B. (2019). Influence of laterality on testis anatomy and histology in Ghezel rams. Veter Med. Sci..

[10] Potter S.J., DeFalco T. (2017). Role of the testis interstitial compartment in spermatogonial stem cell function. Reproduction.

[11] Fayomi A.P., Orwig K.E. (2018). Spermatogonial stem cells and spermatogenesis in mice, monkeys and men. Stem Cell Res..

[12] Ramm S.A., Schaerer L., Ehmcke J., Wistuba J. (2014). Sperm competition and the evolution of spermatogenesis. Mol. Hum. Reprod..

[13] Wanjari U.R., Gopalakrishnan A.V. (2024). Blood-testis barrier: A review on regulators in maintaining cell junction integrity between Sertoli cells. Cell Tissue Res..

[14] Stanton P.G. (2016). Regulation of the blood-testis barrier. Semin. Cell Dev. Biol..

[15] O’Donnell L., Smith L.B., Rebourcet D. (2022). Sertoli cells as key drivers of testis function. Semin. Cell Dev. Biol..

[16] You X., Chen Q., Yuan D., Zhang C.C., Zhao H.X. (2021). Common markers of testicular Sertoli cells. Expert. Rev. Mol. Diagn..

[17] Griswold M.D. (1998). The central role of Sertoli cells in spermatogenesis. Semin. Cell Dev. Biol..

[18] Meroni S.B., Galardo M.N., Rindone G., Gorga A., Riera M.F., Cigorraga S.B. (2019). Molecular mechanisms and signaling pathways involved in Sertoli cell proliferation. Front. Endocrinol..

[19] Griswold M.D. (2018). 50 years of spermatogenesis: Sertoli cells and their interactions with germ cells. Biol. Reprod..

[20] Papadopoulos V., Zirkin B.R. (2021). Leydig cell aging: Molecular mechanisms and treatments. Vitam. Horm..

[21] Shima Y. (2019). Development of fetal and adult Leydig cells. Reprod. Med. Biol..

[22] Svechnikov K., Landreh L., Weisser J., Izzo G., Colón E., Svechnikova I., Söder O. (2010). Origin, development and regulation of human Leydig cells. Horm. Res. Paediatr..

[23] Zhou R., Wu J.R.Z., Liu B., Jiang Y.Q., Chen W., Li J., He Q.Y., He Z.P. (2019). The roles and mechanisms of Leydig cells and myoid cells in regulating spermatogenesis. Cell Mol. Life Sci..

[24] Varga I., Bódi I., Kachlík D., Mestanová V., Klein M. (2019). The enigmatic thymic myoid cells—Their 130 years of history, embryonic origin, function and clinical significance. Biologia.

[25] Neto F.T.L., Phil Vu B., Najari B.B., Li P.S., Goldstein M. (2016). Spermatogenesis in humans and its affecting factors. Semin. Cell Dev. Biol..

[26] O’Shaughnessy P.J. (2014). Hormonal control of germ cell development and spermatogenesis. Semin. Cell Dev. Biol..

[27] White-Cooper H., Bausek N. (2010). Evolution and spermatogenesis. Philos. Trans. R. Soc. Lond. B Biol. Sci..

[28] Dong F., Ping P., Ma Y., Chen X.-F. (2023). Application of single-cell RNA sequencing on human testicular samples: A comprehensive review. Int. J. Biol. Sci..

[29] Papalexi E., Satija R. (2018). Single-cell RNA sequencing to explore immune cell heterogeneity. Nat. Rev. Immunol..

[30] Suzuki T. (2023). Overview of single-cell RNA sequencing analysis and its application to spermatogenesis research. Reprod. Med. Biol..

[31] Huang L., Zhang J., Zhang P., Huang X., Yang W., Liu R., Sun Q., Lu Y., Zhang M., Fu Q. (2023). Single-cell RNA sequencing uncovers dynamic roadmap and cell-cell communication during buffalo spermatogenesis. Iscience.

[32] Wang X., Pei J., Xiong L., Guo S., Cao M., Kang Y., Ding Z., La Y., Liang C., Yan P. (2023). Single-cell RNA sequencing reveals atlas of yak testis cells. Int. J. Biol. Sci..

[33] Wang X., Pei J., Xiong L., Kang Y., Guo S., Cao M., Ding Z., Bao P., Chu M., Liang C. (2023). Single-cell RNA sequencing and UPHLC-MS/MS targeted metabolomics offer new insights into the etiological basis for male cattle-yak sterility. Int. J. Biol. Macromol..

[34] Ren F., Xi H., Qiao P., Li Y., Xian M., Zhu D., Hu J. (2022). Single-cell transcriptomics reveals male germ cells and Sertoli cells developmental patterns in dairy goats. Front. Cell Dev. Biol..

[35] Yu X., Li T., Du X., Shen Q., Zhang M., Wei Y., Yang D., Xu W., Chen W., Bai C. (2021). Single-cell RNA sequencing reveals atlas of dairy goat testis cells. Zool. Res..

[36] Tang X., Chen C., Yan S., Yang A., Deng Y., Chen B., Gu J. (2024). Single-nucleus RNA-seq reveals spermatogonial stem cell developmental pattern in shaziling pigs. Biomolecules.

[37] Zhang L., Guo M., Liu Z., Liu R., Zheng Y., Yu T., Lv Y., Lu H., Zeng W., Zhang T. (2022). Single-cell RNA-seq analysis of testicular somatic cell development in pigs. J. Genet. Genom..

[38] Yang H., Ma J., Wan Z., Wang Q., Wang Z., Zhao J., Wang F., Zhang Y. (2021). Characterization of sheep spermatogenesis through single-cell RNA sequencing. FASEB J..

[39] Wu Y., Guo T., Li J., Niu C., Sun W., Zhu S., Zhao H., Qiao G., Han M., He X. (2022). The transcriptional cell atlas of testis development in sheep at pre-sexual maturity. Curr. Issues Mol. Biol..

[40] Zheng Y., Liu Y., Hou R., Shi K., Chen Y., Feng T., An J. (2022). Single-cell RNA-sequencing analysis and characterisation of testicular cells in giant panda (*Ailuropoda melanoleuca*). Reprod. Fertil. Dev..

[41] Jia R., Huang X.Y., Yang J.J., Wang L.L., Li J., Li Y., Gun S., Yan Z.Q., Wang P.F., Yang Q.L. (2024). Gender-specific DNA methylation profiles associated with adult weight in Hezuo pigs. Int. J. Mol. Sci..

[42] Dobin A., Davis C.A., Schlesinger F., Drenkow J., Zaleski C., Jha S., Batut P., Chaisson M., Gingeras T.R. (2013). STAR: Ultrafast universal RNA-seq aligner. Bioinformatics.

[43] Lun A.T.L., Riesenfeld S., Andrews T., The Phuong D., Gomes T., Marioni J.C. (2019). Emptydrops: Distinguishing cells from empty droplets in droplet-based single-cell RNA sequencing data. Genome Biol..

[44] Butler A., Hoffman P., Smibert P., Papalexi E., Satija R. (2018). Integrating single-cell transcriptomic data across different conditions, technologies, and species. Nat. Biotechnol..

[45] Camp J.G., Sekine K., Gerber T., Loeffler-Wirth H., Binder H., Gac M., Kanton S., Kageyama J., Damm G., Seehofer D. (2017). Multilineage communication regulates human liver bud development from pluripotency. Nature.

[46] Qiu X., Mao Q., Tang Y., Wang L., Chawla R., Pliner H.A., Trapnell C. (2017). Reversed graph embedding resolves complex single-cell trajectories. Nat. Methods.

[47] Guo J., Nie X., Giebler M., Mlcochova H., Wang Y., Grow E.J., Kim R., Tharmalingam M., Matilionyte G., DonorConnect (2020). The dynamic transcriptional cell atlas of testis development during human puberty. Cell Stem Cell.

[48] Figueiredo M.G., Gagliano-Juca T., Basaria S. (2023). Male reproduction and aging. Endocrinol. Metab. Clin. N. Am..

[49] Qu N., Ogawa Y., Kuramasu M., Nagahori K., Sakabe K., Itoh M. (2020). Immunological microenvironment in the testis. Reprod. Med. Biol..

[50] Makela J.-A., Koskenniemi J.J., Virtanen H.E., Toppari J. (2019). Testis development. Endocr. Rev..

[51] Walker W.H. (2009). Molecular mechanisms of testosterone action in spermatogenesis. Steroids.

[52] Wang Y., Navin N.E. (2015). Advances and applications of single-cell sequencing technologies. Mol. Cell.

[53] Zhang L., Li F., Lei P., Guo M., Liu R., Wang L., Yu T., Lv Y., Zhang T., Zeng W. (2021). Single-cell RNA-sequencing reveals the dynamic process and novel markers in porcine spermatogenesis. J. Anim. Sci. Biotechnol..

[54] Choi Y.H., Kim J.K. (2019). Dissecting cellular heterogeneity using single-cell RNA sequencing. Mol. Cells.

[55] Smith L.B., Walker W.H. (2014). The regulation of spermatogenesis by androgens. Semin. Cell Dev. Biol..

[56] Yan Y., Zhu S., Jia M., Chen X., Qi W., Gu F., Valencak T.G., Liu J., Sun H. (2024). Advances in single-cell transcriptomics in animal research. J. Anim. Sci. Biotechnol..

[57] Lukassen S., Bosch E., Ekici A.B., Winterpacht A. (2018). Single-cell rna sequencing of adult mouse testes. Sci. Data.

[58] Xia K., Luo P., Yu J., He S., Dong L., Gao F., Chen X., Ye Y., Gao Y., Ma Y. (2024). Single-cell RNA sequencing reveals transcriptomic landscape and potential targets for human testicular ageing. Hum. Reprod..

