# Supplementary figures and images for: Transcriptional Profiling of Testis Development in Pre-Sexually-Mature Hezuo Pig
Source: Curr Issues Mol Biol. 2024 Dec 29;47(1):10. doi: 10.3390/cimb47010010 (PMC11763623; doi:10.3390/cimb47010010)

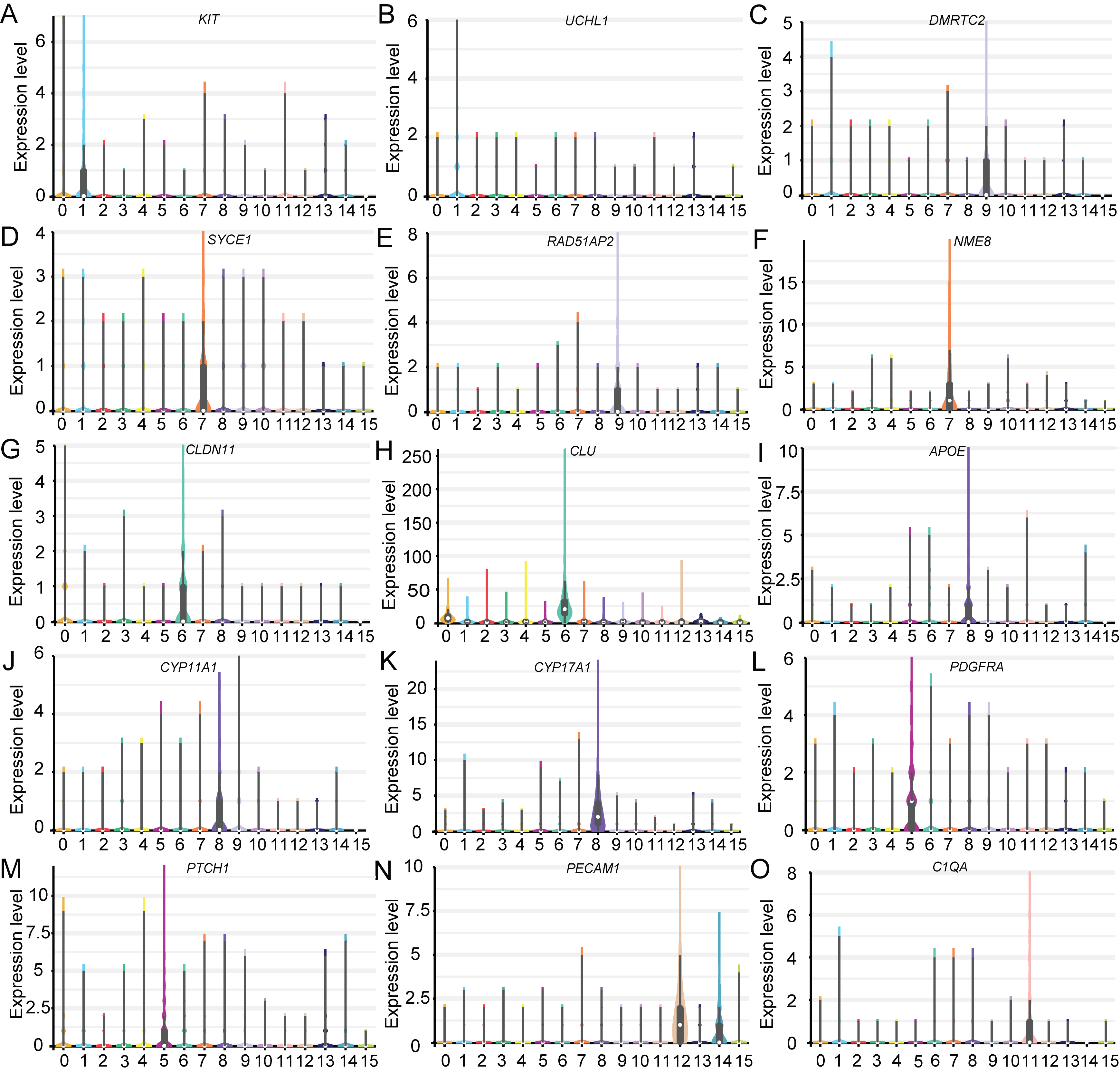

Supplement: Supplementary file 1 [file cimb-47-00010-s001.zip › Supplementary Figure/Figure S1.tif]

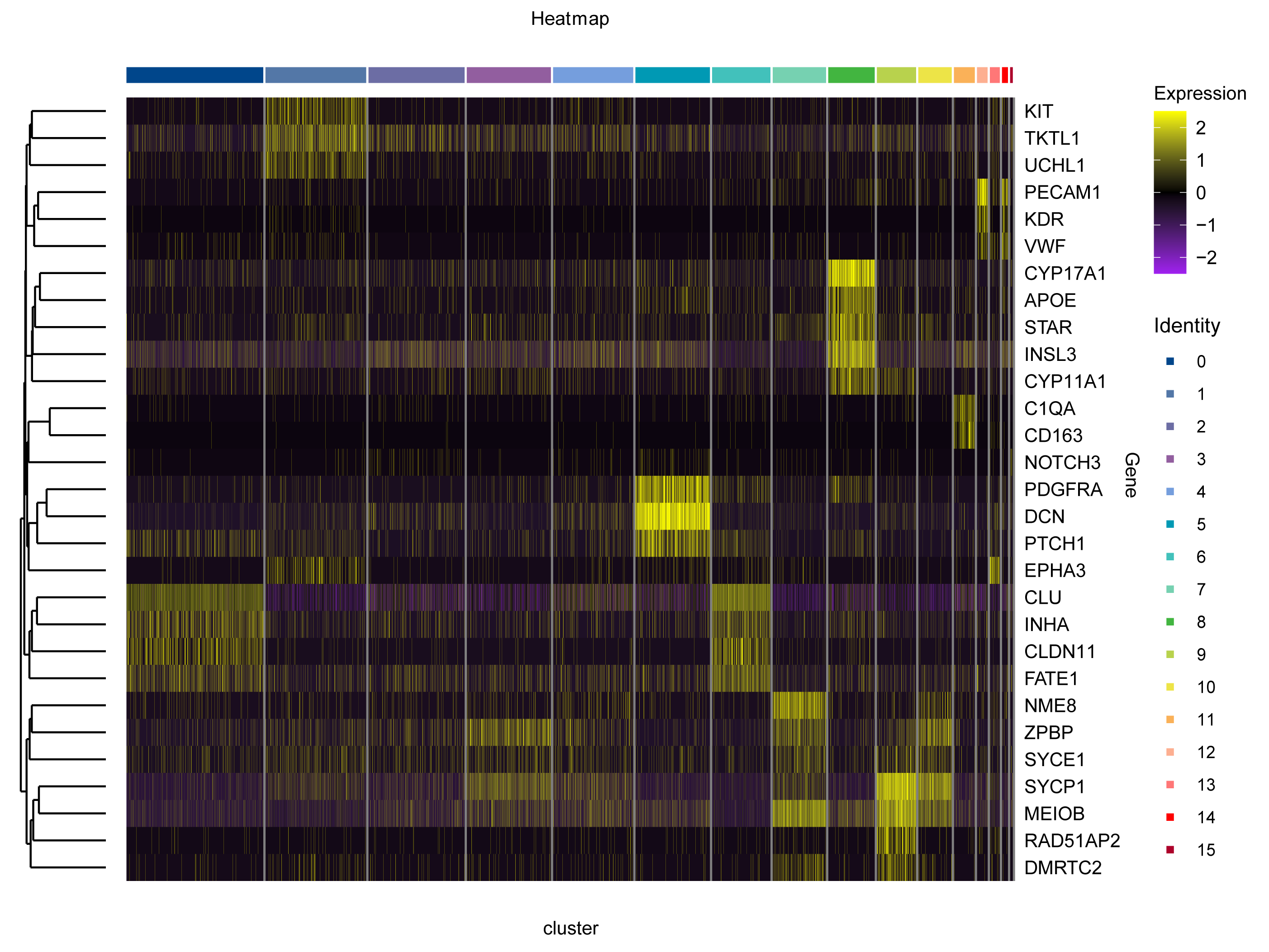

Supplement: Supplementary file 1 [file cimb-47-00010-s001.zip › Supplementary Figure/Figure S2.tif]

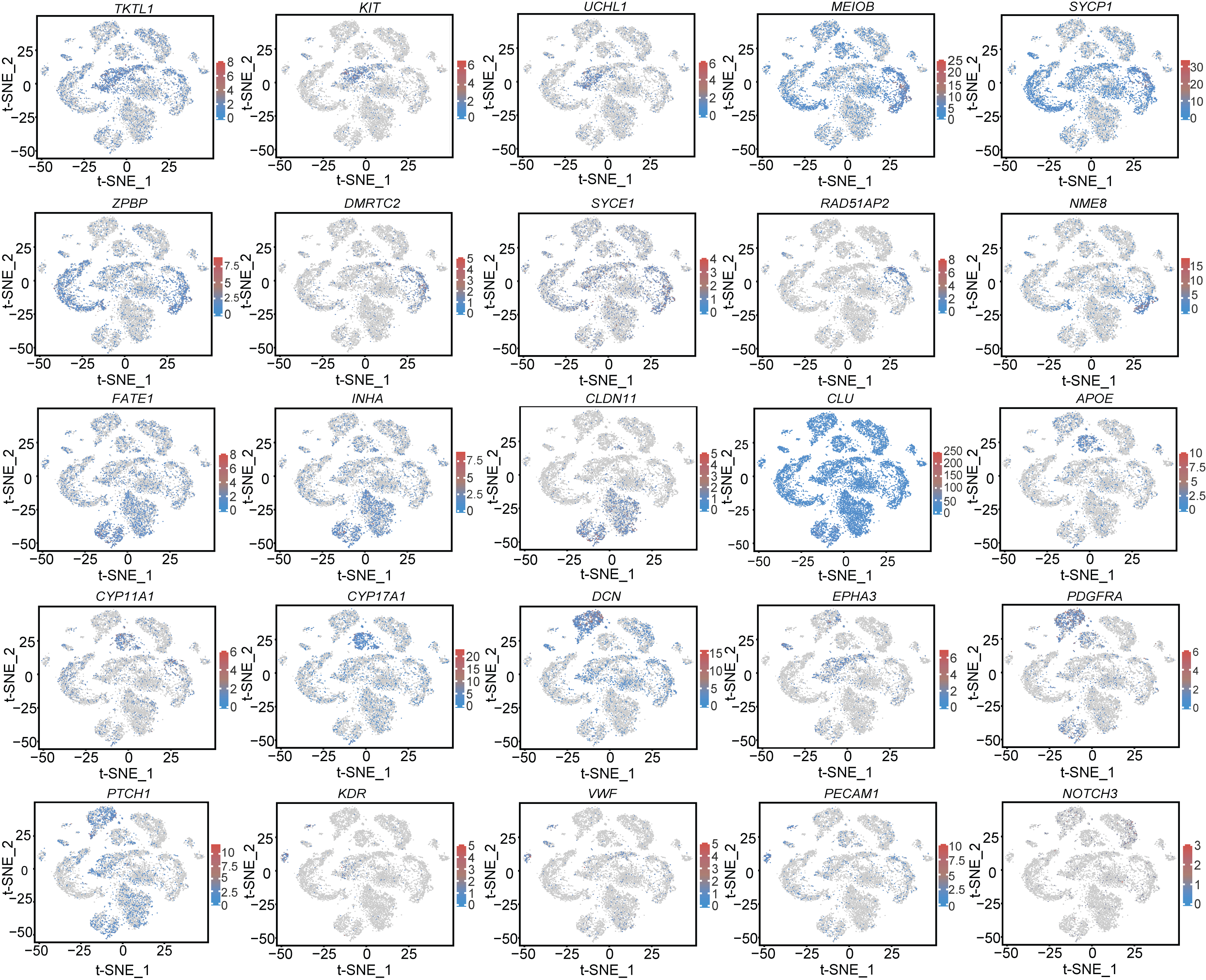

Supplement: Supplementary file 1 [file cimb-47-00010-s001.zip › Supplementary Figure/Figure S3.tif]

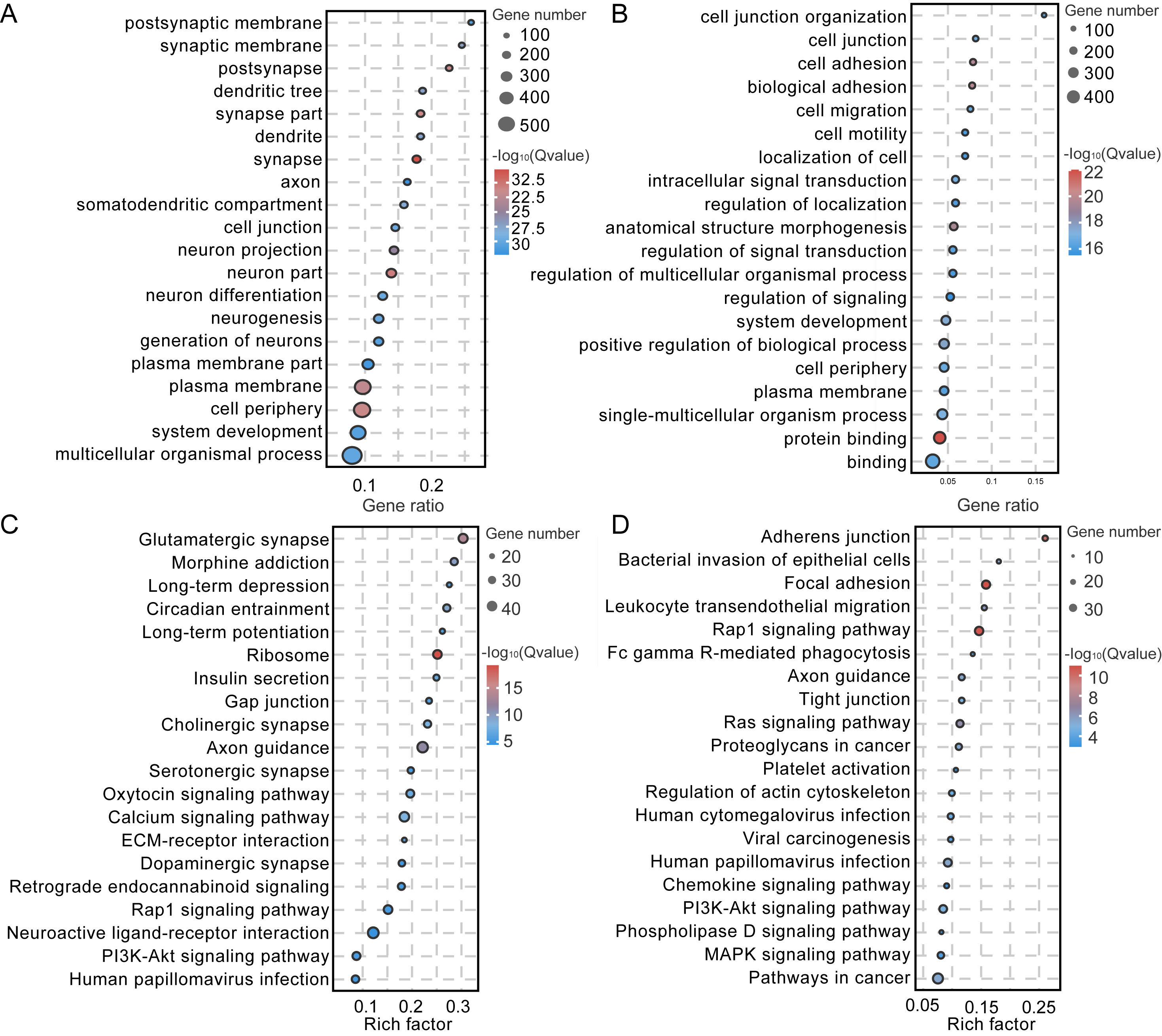

Supplement: Supplementary file 1 [file cimb-47-00010-s001.zip › Supplementary Figure/Figure S4.tif]

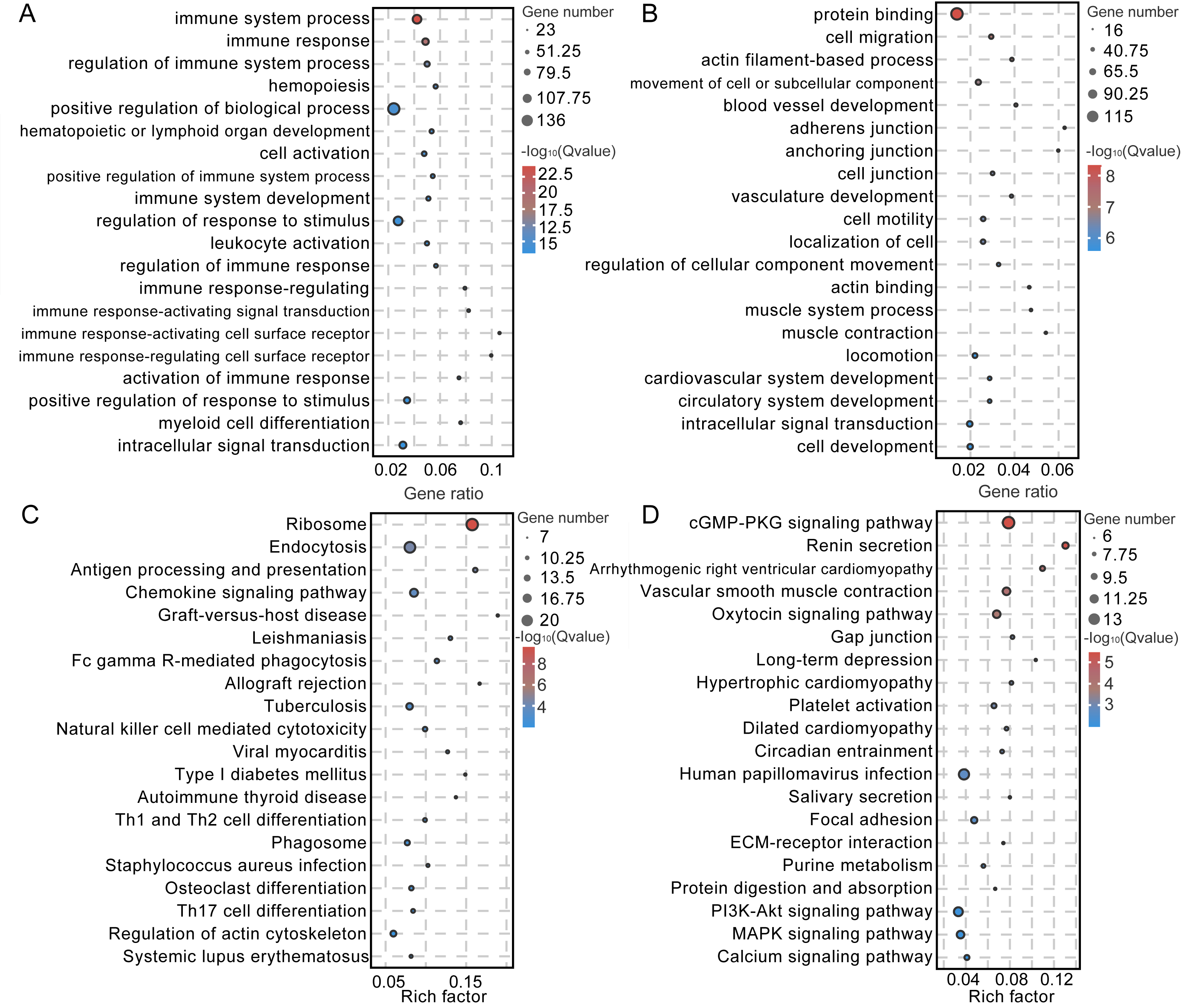

Supplement: Supplementary file 1 [file cimb-47-00010-s001.zip › Supplementary Figure/Figure S5.tif]
